# Supplementary material for: The interactome of intact mitochondria by cross-linking mass spectrometry provides evidence for coexisting respiratory supercomplexes
Source: Mol Cell Proteomics. 2017 Dec 8;17(2):216–32. doi: 10.1074/mcp.RA117.000470 (PMC5795388; doi:10.1074/mcp.RA117.000470)
Supplement: Supplemental Data [file supp_RA117.000470_133922_0_supp_23978_fzffwf.zip › spectra_annotation/mito_spectra_annotation/170-2-3-1-11-1.pdf]

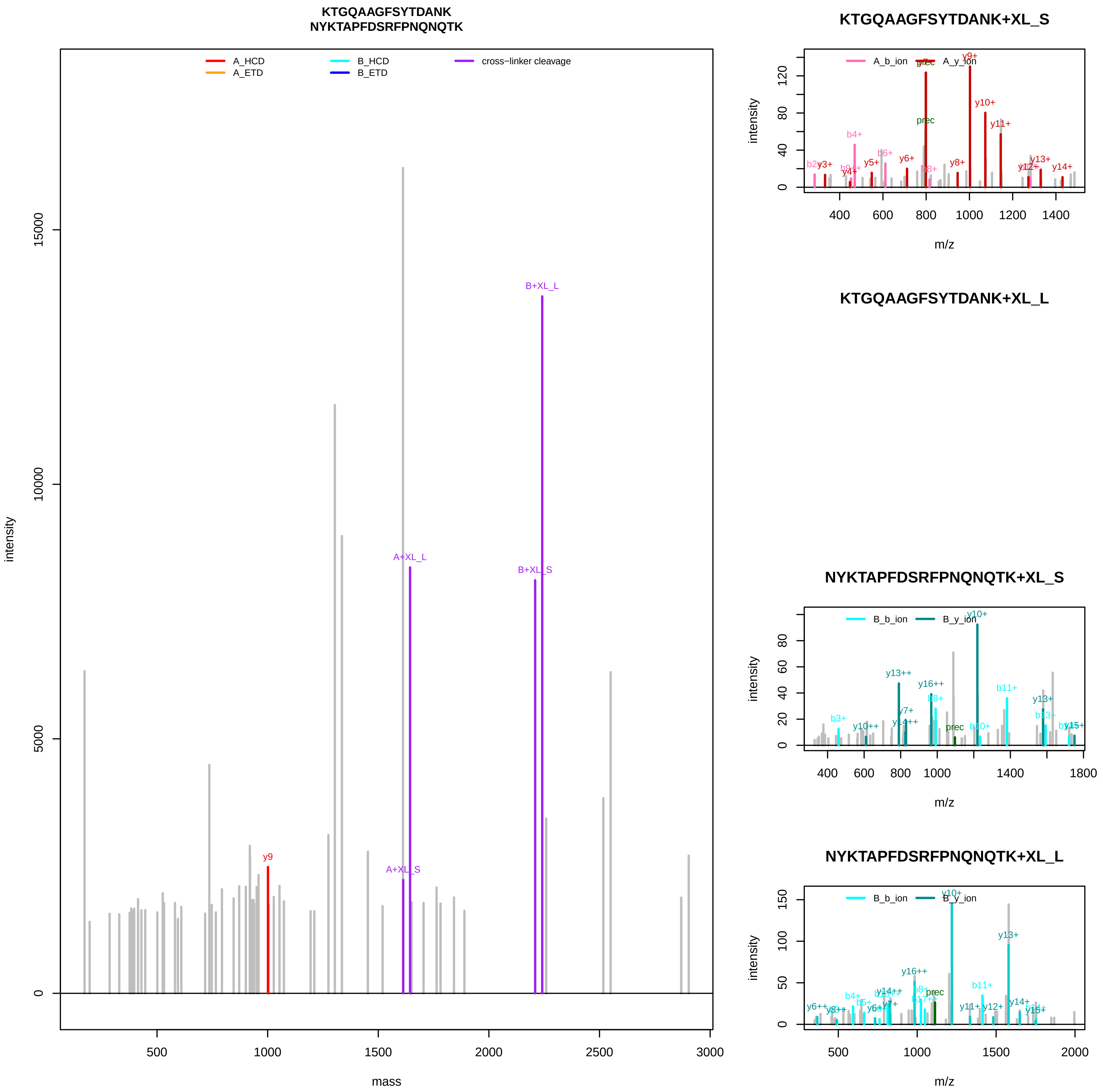
KTGQAAGFSYTDANK
NYKTAPFDSRFPNQNQTK
A_HCD
A_ETD
B_HCD
B_ETD
cross−linker cleavage
B+XL_L
A+XL_L
B+XL_S
A+XL_S
y9
intensity
mass
0
5000
10000
15000
500
1000
1500
2000
2500
3000
KTGQAAGFSYTDANK+XL_S
A_b_ion
A_y_ion
prec
y9+
y10+
y11+
b4+
b6+
y3+
y4+
y5+
y6+
y8+
b8+
y12+
y13+
y14+
m/z
KTGQAAGFSYTDANK+XL_L
NYKTAPFDSRFPNQNQTK+XL_S
B_b_ion
B_y_ion
y10+
y13++
y16++
b8+
b3+
y10++
y7+
y14++
b10+
b11+
y13+
b13+
b15+
NYKTAPFDSRFPNQNQTK+XL_L
y13+
y16++
b11+
b4+
b5+
y6++
y8++
y6+
y7+
y14++
b8+
y11+
y12+
y14+
y15+
